# Identification of novel endogenous antisense transcripts by DNA microarray analysis targeting complementary strand of annotated genes

**DOI:** 10.1186/1471-2164-10-392

**Published:** 2009-08-22

**Authors:** Koji Numata, Yuko Osada, Yuki Okada, Rintaro Saito, Noriko Hiraiwa, Hajime Nakaoka, Naoyuki Yamamoto, Kazufumi Watanabe, Kazue Okubo, Chihiro Kohama, Akio Kanai, Kuniya Abe, Hidenori Kiyosawa

**Affiliations:** 1 Technology and Development Team for Mammalian Cellular Dynamics, BioResource Center (BRC), RIKEN Tsukuba Institute, Ibaraki 305-0074, Japan; 2 Institute for Advanced Biosciences, Keio University, Yamagata 997-0017, Japan; 3 Department of Environmental Information, Keio University, Fujisawa 252-8520, Japan; 4 Experimental Animal Division, BioResource Center (BRC), RIKEN Tsukuba Institute, Ibaraki 305-0074, Japan; 5 C's Lab Co Ltd Maruito Sapporo Bldg, 7F Kita 2 Nishi 1, Kita-ku, Sapporo 060-0002, Japan; 6 Custom Biotechnology Service Group, Hokkaido System Science Co Ltd, 2-1 Shinkawa Nishi 2-1, Kita-ku, Sapporo 001-0932, Japan; 7 Genostaff Inc., Kawauchi Bldg 6F 1-4-4, Nezu, Bunkyo-Ku, Tokyo 113-0031, Japan; 8 Graduate School of Life and Environmental Sciences, University of Tsukuba, Ibaraki 305-0006, Japan; 9 Technology and Development Team for BioSignal Program, BioResource Center (BRC), RIKEN Tsukuba Institute, Ibaraki 305-0074, Japan

## Abstract

**Background:**

Recent transcriptomic analyses in mammals have uncovered the widespread occurrence of endogenous antisense transcripts, termed natural antisense transcripts (NATs). NATs are transcribed from the opposite strand of the gene locus and are thought to control sense gene expression, but the mechanism of such regulation is as yet unknown. Although several thousand potential sense-antisense pairs have been identified in mammals, examples of functionally characterized NATs remain limited. To identify NAT candidates suitable for further functional analyses, we performed DNA microarray-based NAT screening using mouse adult normal tissues and mammary tumors to target not only the sense orientation but also the complementary strand of the annotated genes.

**Results:**

First, we designed microarray probes to target the complementary strand of genes for which an antisense counterpart had been identified only in human public cDNA sources, but not in the mouse. We observed a prominent expression signal from 66.1% of 635 target genes, and 58 genes of these showed tissue-specific expression. Expression analyses of selected examples (*Acaa1b *and *Aard*) confirmed their dynamic transcription *in vivo*. Although interspecies conservation of NAT expression was previously investigated by the presence of cDNA sources in both species, our results suggest that there are more examples of human-mouse conserved NATs that could not be identified by cDNA sources. We also designed probes to target the complementary strand of well-characterized genes, including oncogenes, and compared the expression of these genes between mammary cancerous tissues and non-pathological tissues. We found that antisense expression of 95 genes of 404 well-annotated genes was markedly altered in tumor tissue compared with that in normal tissue and that 19 of these genes also exhibited changes in sense gene expression. These results highlight the importance of NAT expression in the regulation of cellular events and in pathological conditions.

**Conclusion:**

Our microarray platform targeting the complementary strand of annotated genes successfully identified novel NATs that could not be identified by publically available cDNA data, and as such could not be detected by the usual "sense-targeting" microarray approach. Differentially expressed NATs monitored by this platform may provide candidates for investigations of gene function. An advantage of our microarray platform is that it can be applied to any genes and target samples of interest.

## Background

There is a growing body of evidence that natural antisense transcripts (NATs) play important regulatory roles in various biological processes. NATs are usually transcribed from the opposite strand of a particular gene locus, and they are thought to regulate sense gene expression [1,2]. One of the proposed models of NAT-mediated regulation is for the antisense transcript to act as a *cis*-repressor of gene expression from the sense strand. For example, in early embryogenesis, transcription of the antisense genes *Tsix *and *Air *determines the fate of expression of their sense partners *Xist *and *Igf2r*, respectively [3,4]. The appearance of NATs within several imprinted loci suggests that NATs may regulate gene expression by controlling the epigenetic status of surrounding genes [5-7]. Moreover, NATs may function in pathological conditions by causing epigenetic alterations such as histone modification and DNA methylation [8,9].

The other primary model of NAT-mediated gene regulation is induction of the production of small RNAs from NAT loci and their subsequent function in RNA interference (RNAi) pathways. Endogenous small interfering RNA (endo-siRNA) molecules, generated from NAT loci, are induced specifically under conditions of salt stress and immune response in plants [10-15]. Recent experimental data also suggests the presence of NAT-associated endo-siRNA molecules in animals [16-18].

Although the number of NATs thought to have biological functions has gradually increased, the functions of most NATs discovered in recent large-scale *in silico *studies are unknown. Computational identification of NATs is based mostly on the analysis of cDNA and EST sequence collections by sequence alignment, and this process has identified several thousand sense-antisense pairs [19]. However, in principle, cDNA sequencing accumulates data on transcripts with poly(A)-stretches and does not access the non-poly-adenylated population of transcripts. A recent genome-wide tiling array study of the human genome revealed that many genomic regions that could not be identified from cDNA collections are apparently transcribed and tend not to be poly-adenylated [20]. This finding indicates that antisense transcriptome analyses based solely on cDNA information may be inefficient. In addition, most publicly available cDNA sequences are derived from normal cellular conditions, such as normal adult tissues, and thus are not useful for the identification of NATs specific to abnormal cellular conditions.

To discover novel NATs expressed under various biological conditions, we proposed a microarray-based technique involving the use of 60-mer oligonucleotide DNA probes selected from the complementary sequences of cDNAs (*i.e*., known genes), referred to as artificial antisense sequence (AFAS) probes. This approach has the ability to detect antisense expression that cannot be identified by using information from the cDNA and EST collections and has the advantage of compatibility with the computational methodology widely used for sense gene expression analysis [21]. We performed microarray analyses with AFAS probes by using oligo-dT and random primed target samples to provide a comprehensive approach for the detection of novel non-poly-adenylated transcripts in the antisense transcriptome.

Here, we designed AFAS probes to correspond to the antisense strand of well-studied selected genes, including oncogenes and tumor suppressor genes, imprinted genes, and human-mouse orthologous genes. We studied the expression profiles of targeted transcripts in normal mouse adult tissues and in mouse mammary tumor virus (MMTV)-induced mammary tumors. This technique is applicable to all genes and sample types and can be used for antisense expression identification that is not possible by using conventional cDNA information alone.

## Results

### AFAS probes detect previously known NATs

**Figure 1:**
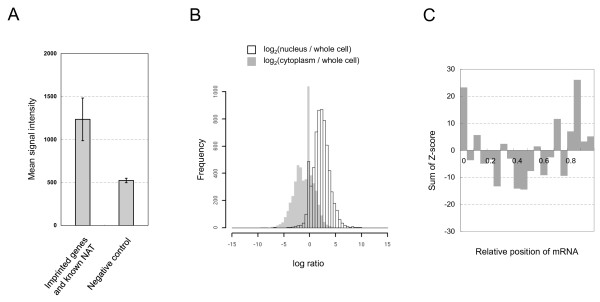
**Global tendency of AFAS probes in normal tissues**. (**A**) Mean signal intensities of AFAS probes were compared between sets of known NAT-associated genes (including imprinted genes) and negative controls. Negative controls with no cDNA, EST, and CAGE tags in their antisense orientation were randomly picked from NCBI RefSeq. (**B**) Nuclear enrichment (grey bars) was measured by log_2_(S_nucleus_/S_whole-cell_), where S_nucleus _and S_whole-cell _denote signals from nuclear fractions and from whole cells (NIH3T3), respectively. Cytoplasmic enrichment (open bars) was calculated by log_2_(S_cytoplasm_/S_whole-cell_), where S_cytoplasm _denotes signals from the cytoplasmic fraction. All signal intensities were obtained from the experiments by using random-primed samples. (**C**) The sum of Z-scores for every relative position is indicated. For each tissue, the positional preference of NAT expression was measured by Z-scores calculated from the median signal values of every position. Expression data were obtained from random-primed samples.

To verify whether our methods can detect NAT expression, we initially examined the signal intensities of AFAS probes that targeted previously identified antisense transcripts. For example, AFAS probes designed for *Tsix*, which reflects the abundance of *Xist *RNA, detected expression in the 11 adult mouse tissues (mixed males and females), but not in the testis, as expected (see Additional file 1). Such expression patterns were detected only for probes corresponding to the exonic-overlapping regions between *Tsix *and *Xist*, and also for the sense probes corresponding to *Xist *RNA (see Additional file 1). This finding suggests that AFAS probes for *Tsix *can identify not only the presence of its antisense counterpart, but also its exonic regions. In addition, AFAS probes for several imprinted genes (*Igf2r*, *Kcnq1*, *Gnas*, *Dio3*, and *Ube3a*), which are known to give rise to antisense transcripts [5], also gave prominent signals (see Additional file 2). Moreover, previously known antisense transcripts, such as those arising from *Myc *(myelocytomatosis oncogene) [22] and *Tgfb2 *(transforming growth factor beta2) [23], were also detected by our microarray platform (data not shown). Although the number of documented NAT examples in normal mouse adult tissues is limited, the mean signal intensities generated by AFAS probes corresponding to these genes were higher than those for the negative control genes. The control genes comprise a set of randomly selected genes, without cDNA, EST, and CAGE tags in the antisense orientation (Figure 1A, *P *= 5.1e-12 by Welch's t-test). These data indicate that endogenous NAT expression of known genes is potentially detectable by probes designed for the complementary strand of known genes.

### Global analyses of AFAS probes

Before screening for novel NATs using AFAS probes, we first analyzed the global tendency of signal intensities from all AFAS probes applied to our custom microarray platform. Because Northern blot analyses for particular gene loci have previously shown that NATs tend to be poly(A)-negative [24], we checked whether our AFAS probes also showed this tendency in normal mouse tissue expression profiling. A significantly higher number of AFAS probes than sense probes detected transcripts only within random-primed samples, but not among the oligo-dT primed targets (*P *< 2.2e-16, Fisher's exact test, see Additional file 3). This result indicates that transcripts detected by AFAS probes also lack poly(A)-tails, similar to the finding for NATs characterized by Northern blot analyses [24]. Also, the number of sense probes detecting transcripts in both oligo-dT and random primed samples was higher than that of antisense probes (*P *< 2.2e-16, Fisher's exact test, see Additional file 3). This finding indicates that sense transcripts with poly(A)-tails can be identified by both priming methods, because sense probes target the protein-coding strand of the mRNA, which is expected to have a poly(A)-tail. Another characteristic of endogenous NATs is their nuclear localization [24]. A distribution comparison of AFAS probe signals between nuclear and cytoplasmic fractions clearly showed nuclear enrichment of detected transcripts (Figure 1B, *P *< 2.2e-16, Median test).

Several large-scale studies using information in cDNA and EST collections and from genome-wide tiling arrays in yeast previously showed that NATs tend to be transcribed from the 3' region of its counterpart mRNA [25-27], thus implying the presence of regulatory mechanisms involving tail-to-tail overlapping. We also observed this characteristic for the AFAS probes, because the AFAS probe signals clearly showed positional preference relative to the sense mRNA (Figure 1C). This result indicates that AFAS probes indeed detect the positional bias of antisense transcription. Similarly, we also observed higher signals within 5' regions (Figure 1C), thus suggesting that NATs may also arise near the transcriptional start site, as previously shown for head-to-head overlapping NATs such as *WT1*, *Sphk1*, and *Tsix *[28-30].

### Novel conserved NAT detection by normal tissue profiling

**Figure 2:**
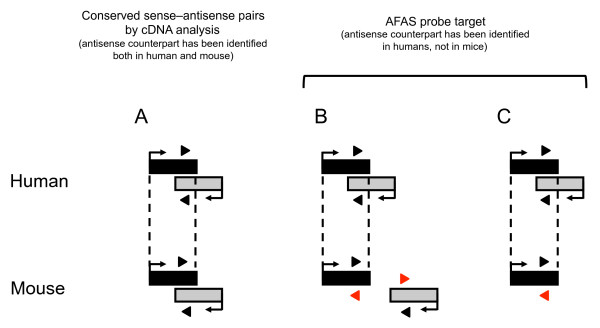
**Schematic illustration of the strategy to discover novel conserved NATs**. Conserved NATs were previously listed according to the presence of cDNA within humans and mice (**A**). Black and grey boxes denote transcribed region identified by cDNA on plus and minus strand of the genome, respectively. Black triangles indicate microarray probes designed within the transcribed regions. If antisense counterpart on opposite strand has not been overlapped, or not been identified in mice (**B and C**), microarray probes attempted to detect antisense expression could not be designed. To discover novel conserved NAT, we designed AFAS probes (red triangles) for mouse genes for which the orthologous partner in the human genome has an antisense counterpart.

To test the ability of AFAS probes to detect novel NATs, we initially applied our microarray approach to the human-mouse orthologous gene set. In many studies, inter-species conservation of NATs is implied by the presence of common cDNA sequences between the two species [26,31,32]. However, recent genome-wide tiling array and CAGE analyses revealed that a large fraction of the genome is transcribed [33,34], indicating that current cDNA collection is not sufficient for comprehensive comparative genomics, including comparative antisense transcriptome analyses. In this situation, the use of AFAS probes corresponding to genes for which the antisense counterpart has been identified in humans, but not in mice, may lead to the detection of novel conserved NATs (Figure 2).

We designed AFAS probes corresponding to 635 mouse orthologous partners, for which the antisense counterpart has been identified in humans, but not in mice (one sense and one antisense probe were designed per gene). We then profiled the expression of these genes to detect antisense expression within 12 normal mouse tissues. We identified 420 (66.1%) probes that gave a signal (signal intensity ≥100, which is our empirically defined criterion), at least in a single particular tissue, and 58 of these (9.2%) showed tissue-specific expression. Probes of 120 genes gave signals with a higher than average intensity according to inter-array normalization (see Additional file 4). These results suggest that many NATs identified only in the human cDNA collection may also be expressed in mice.

We attempted to validate the expression of two candidate conserved NATs (antisense of *Acaa1b *and *Aard*) by performing Northern and *in situ *hybridization (ISH) analyses. Whereas human *ACAA1 *(acetyl-Coenzyme A acyltransferase 1) overlaps with *DLEC1 *(deleted in lung and esophageal cancer 1) in a tail-to-tail overlapping manner, its orthologous counterpart in the mouse genome (*Acaa1b *and *Dlec1*) shows a tail-to-tail relationship but not a reciprocal overlapping relationship, according to the annotated gene structure (see Additional file 5). Both microarray and Northern analyses confirmed that the *Acaa1b *sense transcript is expressed within liver and kidney (see Additional file 5). Northern analyses were not able to detect the antisense transcript of *Acaa1b *from either poly(A)+ or total RNA (data not shown), but quantitative RT-PCR, ISH and microarray analyses were able to detect this transcript within the testis and kidney (see Additional file 5). This result implies that NATs detected by microarray analysis using AFAS probes are transcribed *in vivo*.

**Figure 3:**
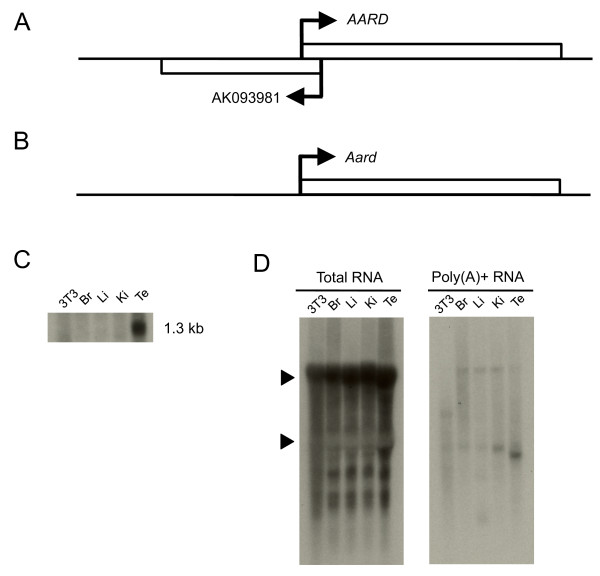
**Northern analyses of the sense and antisense transcripts of *Aard***. (**A**) Schematic illustration of *AARD *and AK093989 (unannotated gene product) in humans. (**B**) Mouse orthologous partner, *Aard*. (**C**) Northern analysis for sense expression of *Aard *in NIH3T3 cells, brain (Br), liver (Li), kidney (Ki), and testis (Te). (**D**) Northern analysis for expression of the corresponding antisense transcript for both poly(A)+ and total RNA. Triangles indicate 28S (4710 nt) and 18S (1870 nt) ribosomal RNA.

**Figure 4:**
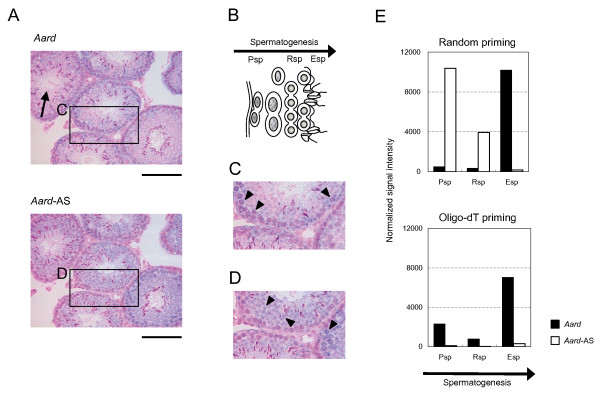
**Expression dynamics of *Aard *and *Aard*-AS**. (**A**) *In situ *hybridization results (seminiferous tubules) for sense (*Aard*) and antisense (*Aard*-AS) transcripts. Arrow indicates the direction of spermatogenesis, as illustrated in (**B**). Scale bars: 100 μm. (**C and D**) Enlargement of the corresponding boxes in (**A**). Arrowheads denote the cytoplasmic signal of *Aard *and the nuclear signal of *Aard*-AS. (**E**) Microarray results of *Aard *and *Aard*-AS expression during the spermatogenesis. Black and white bars indicate normalized signal intensity levels of sense and AFAS probe, respectively. Arrow indicates the direction of spermatogenesis (Psp, pachytene spermatocytes; Rsp, round spermatids; Esp, elongated spermatids). Fractionation of germ cells on the basis of the three stages of spermatogenesis in the mouse testis was performed as previously described [53,54].

We also analyzed the expression of *Aard *(alanine- and arginine-rich domain-containing protein), which is a functionally uncharacterized gene but is known to be expressed within the adult testis and XY fetal gonad [35]. In humans, exons of *AARD *(also known as C8orf85) overlap with that of an unnamed uterus EST (GenBank: AK093981), whereas mouse *Aard *has no EST arising from the antisense strand (Figure 3B). Northern analysis confirmed that expression of the sense transcript of *Aard *was testis-specific (Figure 3C); however, Northern analysis of the antisense transcript showed laddered hybridization patterns for total RNA, but not for poly(A)+ RNA isolated from all samples (Figure 3D). By comparison, both the sense and antisense transcripts (*Aard*-AS) were detected by ISH within a particular region of the seminiferous tubules (Figure 4A,B), thus confirming that the *Aard*-AS is also expressed in the testis. In addition, *Aard*-AS was most likely located within the nucleus, whereas *Aard *was located within the cytoplasm (Figure 4C,D). Because ISH shows that *Aard*-AS is expressed in a particular region of the seminiferous tubules, we checked our microarray data on fractionated testis samples that reflected the three steps of spermatogenesis (*i.e*., pachytene spermatocytes, round spermatids, and elongated spermatids). We found that *Aard*-AS was expressed within the early period of spermatogenesis, whereas the sense transcript appeared at a later phase (Figure 4E). This finding shows that sense and antisense transcripts of *Aard *are transcribed exclusively and in a mutually antagonistic fashion during spermatogenesis. In addition, *Aard*-AS expression was detected only in the random-primed target sample, not in the oligo-dT primed target (Figure 4E), indicating that *Aard*-AS tends to be poly(A)-negative and nuclear-localized.

These data clearly confirm that AFAS probes can detect the expression of antisense transcripts in normal tissues, and that they can also identify transcripts expressed in a tissue- and cell-type-specific manner. Detection of such expression dynamics for antisense transcripts is possible only by using the analytical platform targeting the complementary strand of the annotated genes. Thus, AFAS probes, when used within appropriate biological samples and combined with other analytical modalities, can be used to discover genuine functional NATs; this is an advantage over conventional approaches that depend on publicly available cDNA data.

### Detection of novel NATs differentially expressed under pathological conditions

We next checked whether AFAS probes have the ability to detect antisense transcripts in cancerous tissues. Examples of functional antisense transcripts identified in abnormal cells are *CDKN2B*, *WT1*, and *HBA2 *[8,9,29]. These antisense transcripts control the epigenetic status of surrounding genes by DNA methylation or histone modification and thus are thought to affect the expression of their sense partners. To confirm this notion, we applied the AFAS probe technique to the 404 well-characterized genes including oncogenes and tumor suppressors (1752 AFAS probes were successfully designed, giving 4.4 probes per gene on average). We used these probes in microarray experiments based on the GRS/A mouse strain, which frequently suffers from (MMTV)-induced mammary tumors [36].

**Figure 5:**
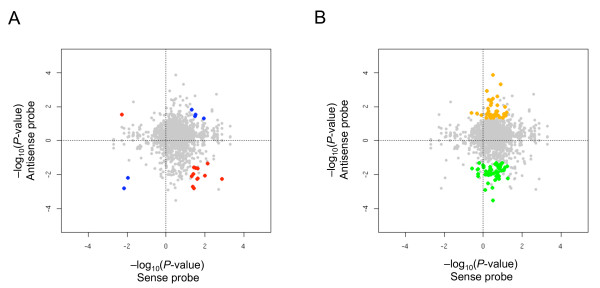
**Probes showing differential expression between normal and tumor regions**. Differential expression between normal and tumor regions is plotted with log-scaled *P *value according to Student's *t*-test for sense (x-axis) and antisense (y-axis) expression. In cases where the mean value of signals from normal regions was higher than that of tumors, the value was multiplied by -1. Accordingly, values higher than zero indicate up-regulation, whereas values lower than zero indicate down-regulation, in tumors. (**A**) Colored points denote significant changes in the expression of both the sense and antisense transcripts in tumors, in a correlated manner (blue) and in an anti-correlated manner (red). The names of the genes indicated by the colored points are listed in additional file 6. (**B**) Orange and green dots indicate up-regulated and down-regulated antisense expression from genes, respectively, but no apparent changes in sense transcript expression.

For the probes designed to detect the sense transcripts, we identified 57 genes showing differential expression. Among these, 48 were up-regulated and 9 were down-regulated within tumor regions, compared with in normal regions, according to a set statistical threshold (*P *≤ 0.05 by Student's *t*-test) (Figure 5 and Additional file 6). Among the up-regulated genes in tumors, 12 genes (*Pdcd6 *is shown as an example in Additional file 7) showed loss of antisense expression (Figure 5A, right lower), whereas among the down-regulated genes *Nr2c2 *showed up-regulation of its antisense expression in an anti-correlated manner with the sense transcript counterpart (Figure 5A, left upper). These genes are reminiscent of the model in which antisense transcription may lead to the silencing of sense gene expression, such as cyclin-dependent kinase inhibitor (*CDKN2B*) and its antisense counterpart [9]. These genes may be regulated through an antisense-mediated pathway.

**Figure 6:**
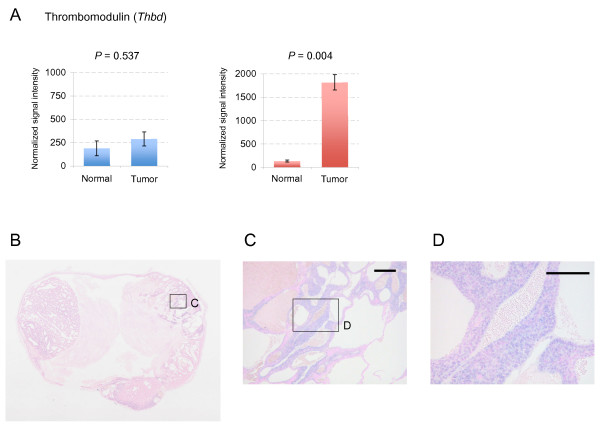
***In situ *hybridization of the antisense transcript of *Thbd***. (**A**) Microarray results for *Thbd*, for which expression of the antisense transcript (red bars) has markedly changed in tumor cells but that of the sense transcript (blue bars) has not. (**B-D**) Results of *in situ *hybridization of mammary tumor tissue of GRS/A mice for detecting antisense transcription of *Thbd*. Scale bars: 200 μm (**C**); 100 μm (**D**).

Interestingly, the expression of antisense transcripts representing 37 genes (*Thbd *is shown as an example in Figure 6A) was found to increase, despite the absence of changes in expression of their sense transcript counterparts (Figure 5B). We also identified down-regulated antisense transcripts corresponding to 45 genes (*Drd4 *is shown as an example in Additional file 7) for which there were no changes in expression of their corresponding sense transcripts. Because ISH using cancerous tissues, like microarray analysis, can detect antisense expression arising from *Thbd *(thrombomodulin) (Figure 6B–D), there might be more examples of genes for which antisense expression is altered in cancerous tissue but cannot be detected by microarray analysis that targets expression from the sense strand of genes.

## Discussion

This paper shows that microarray probes targeting transcription from the complementary strand of known genes can identify novel NATs, an approach that has not been possible solely on the basis of publicly available cDNA data. Recently described high-density oligonucleotide tiling-array platforms are designed to overview the transcriptional landscape of specific genomic regions at high resolution. By comparison, our platform uses multiple probes to specifically screen for transcription from the antisense strand of known genes. Many previous studies have attempted to identify NATs by DNA microarray analysis using cDNA-oriented custom microarrays or commercially available microarray platforms [37-41]. Since our microarray platform is custom-made and not commercial, it can be applied to any genes or gene loci of interest. Furthermore, our method does not introduce bias from cDNA synthesis between sense and antisense profiling because it does not require specific protocols for target cDNA synthesis for NAT detection. In addition, our microarray platform approach can simultaneously profile sense and antisense expression in one microarray hybridization experiment.

Many NATs detected by AFAS probes were appeared only in the random-primed targets. This was concordance with previous cDNA-based microarray profiling of NAT expression [24]. Whereas poly(A)-plus RNA population is roughly represented by oligo-dT primed cDNAs, whole transcriptome (including the poly(A)-minus RNA population) is represented by cDNAs synthesized by random primers. Therefore, NATs detected by our analysis tend to be poly(A)-negative. Although oligo-dT primers can pick the internal poly(A)-stretches, this is not an issue at the level of microarray-based NAT screening, because the vast majority of the poly(A)-stretch (approximately 90%) is located within the 3' end of the transcripts (data not shown).

By designating AFAS probes to human-mouse orthologous genes, we identified many probes showing positive signals. Two of these probes identified transcripts for which *in vivo *expression was confirmed. Thus, our approach may reveal more, as yet unidentified, conserved NATs; this has not been possible by conventional approaches, as previously reported using cDNA data [26,31,32]. Of the individually validated examples (*Acaa1b *and *Aard*), expression of *Aard*-AS was localized to the nucleus and was detected only in random-primed target samples. In addition, multiple-size hybridized bands pattern was observed especially for total RNA membrane, not for poly(A)+ RNA membrane. This observation is similar to that of previously identified antisense transcripts [24], and this is probably due to heterogeneously sized molecules of *Aard*-AS transcripts. Because ISH and the microarray data on other antisense transcript examples also show nuclear localization and poly(A)-avoidance (data not shown), it is possible that these features are general characteristics of the antisense transcriptome.

We also designed AFAS probes for well-characterized genes and identified several examples of correlated and anti-correlated expression between the NATs and the corresponding sense transcript within MMTV-induced mammary tumors. We observed differentially expressed genes for which expression of the antisense transcript had changed, whereas that of the sense transcript had not. Given that differential antisense expression might induce changes in epigenetic status, for example in *CDKN2B *and *CDKN2BAS *[9], antisense transcription may cause changes in the methylation status of neighboring genes. This notion can be tested by using methylated DNA immunoprecipitation (MeDIP) and chromatin immunoprecipitation (ChIP) on chip analyses to further characterize the antisense transcriptome and to determine whether specific NATs function as epigenetic regulators. Whereas this study revealed NATs specific to mouse tumors, human clinical samples have also been analyzed to screen for novel NATs by the same methodology; this new study has identified many antisense transcripts showing increased or decreased expression in human colon cancer tissues compared with controls (Saito R., Kohno K., Okada Y., Osada Y., Numata K., Watanabe K., Nakaoka H., Yamamoto N., Kanai A., Yasue H. et al., manuscript in preparation).

Although next-generation high-throughput transcriptome sequencing (RNA-seq) might replace microarray-based expression analyses, antisense transcriptome analysis by sequencing is still under development because of the laborious nature of strand-specific library construction [42]. DNA microarray-based profiling makes it possible to gain a detailed view of specific genes or gene loci and can also provide expression profiles of both poly(A)-plus and poly(A)-minus RNAs.

## Conclusion

We showed here that probes targeting the complementary strand of the annotated genes successfully identify novel NAT expression, including those altered tissue- and tumor-specifically. The results suggest that there are more examples of NATs that cannot not be collected from public cDNA sources. Further functional investigation is required for such dynamically expressed NATs, and the use of microarray platforms targeting both strands of the gene locus will help to narrow down the proper candidates for further functional analyses.

## Methods

### Custom microarray construction

The AFAS probes for detecting NATs were designed to detect antisense transcription originating from genes categorized into three groups: (1) 48 genes in which antisense transcription has been previously reported and 87 imprinted genes in mice, (2) 404 selected well-annotated genes, (3) orthologous genes in NAT loci (detailed definition given below), and (4) randomly selected genes for which there were no cDNA, EST, and CAGE tags in the antisense orientation. For categories (1) and (2), the AFAS probes were designed to correspond to every 500 bases of the antisense strand of the exonic regions of each gene. For category (3), the AFAS probes were designed to correspond to a single specific sequence in each transcript. For category (4), two AFAS probes were designed per transcript. Target region selection for the probe design is summarized in Additional file 8. All probes were computationally designed by using the OligoWiz program [43] and were used in the Agilent 44K custom oligoarray platform for single-color microarray analysis.

### Target sample preparation for the microarray analysis

Total RNA for the mouse (C57BL/6J) microarray experiments was isolated from NIH3T3 cells (fibroblast cell line), SL10 cells (fibroblast cell line), brain, heart, intestine, kidney, liver, lung, placenta (d.p.c. 10.5 and 13.5), spleen, stomach, testis, and thymus. Testis was from C57BL/6J males (8 to 10 weeks), placenta was from pregnant mice, and the other tissue was from both male and female mice. Nuclear and cytoplasmic fractionation of NIH3T3 cells was carried out according to the Protein and RNA Isolation System (PARIS) instructions (Ambion Inc.). For the microarray analysis of murine mammary tumors, RNA samples were collected from normal and cancerous mammary glands of dissected GRS/A mice [36].

### Data processing and the accessibility

Numerical processed signal values (gProcessedSignal) of the Agilent Feature Extraction File were obtained as representative expression levels for each probe within the array. If a spot had an intensity value lower than five, or if there was no prominent difference between foreground and background signals, then the intensity value was adjusted to five and the corresponding probe was treated as an "absent probe". To perform normalization of signal intensity distribution between multiple arrays, the whole mean signal of every hybridization experiment was adjusted to that of the data from SL10 cells by oligo-dT priming. Probes with intensity values lower than five, as well as being flagged as "saturated", were discarded for the inter-array-normalization step. Tissue-specificity of the expression signals was evaluated according to τ measurement [44]. The raw data from the microarray analyses were deposited in the NCBI Gene Expression Omnibus (GEO) under accession number GSE14568 [45]. Expression data as well as a simplified genomic structure can be accessed via an originally constructed viewer [46].

### *In silico *identification of orthologous genes in NAT loci

To identify orthologous genes in NAT loci (Figure 2), we initially performed *in silico *identification of sense-antisense pairs by the same procedures as previously published [47], by using the latest full-length cDNA collections [33,48], NCBI RefSeq mRNA [49] and the UniGene collection [50]. This identified 3524 and 5351 exon-overlapping sense-antisense pairs in humans and mice, respectively. Genomic synteny data between human and mouse (defined by BLASTZ derived from UCSC [51]) was then exploited to determine whether each identified pair was located within the syntenic region between the two species. Those pairs located within the syntenic regions were retained for the orthologous relationship validation. The orthologous relationship between the genes located within the syntenic regions was defined according to the orthologous gene table from the BioMart Project [52]. Finally, 648 genes are identified as orthologous genes for which NAT was identified in human cDNAs but not in mouse cDNAs. AFAS probes for these (635 of 648) were successfully designed.

### Northern hybridization analyses

RNA from mouse tissues (C57BL/6J, 8 to 10 weeks, male and female mixed), and the NIH3T3 was isolated by using Trizol reagent (Invitrogen Corporation). Northern analyses were performed as previously described [24]. Loading of equal amounts of RNA samples was confirmed by visualization of ethidium bromide-stained RNA in the gel. Probes specific for sense and antisense of *Acaa1b *(NM_146230), *Aard *(NM_175503), and *Thbd *(NM_009378) were amplified by the PCR (see Additional file 9). All the probe sequences contained their corresponding microarray probe sequences. cDNA fragments were cloned to the pGEM-T Easy Vector (Promega Corporation), and strand-specific cRNA was prepared for hybridization.

### *In situ *hybridization

Probes specific for sense and antisense of *Acaa1b *(NM_146230), *Aard *(NM_175503), and *Thbd *(NM_009378) were amplified by the PCR (see Additional file 9). All the probe sequences contained their corresponding microarray probe sequences. The amplified fragment was sub-cloned into pGEMT-Easy vector (Promega) and was used for generation of sense or antisense RNA probes. Paraffin-embedded testis sections (6 μm) of normal adult mouse (C57BL/6 mouse, male, 8 weeks) were obtained from Genostaff Co., Ltd. For *in situ *hybridization the sections were hybridized with digoxigenin-labeled RNA probes at 60°C for 16 h. The bound label was detected using NBT-BCIP, an alkaline phosphate color substrate. The sections were counterstained with Kernechtrot (Muto Pure Chemicals Co., Ltd.). Probe sequence of negative control experiment was selected from *Oryza sativa *putative leaf protein (NM_197207) (see Additional file 5 and 10).

### Real-time quantitative RT-PCR

cDNA was initially synthesized with gene-specific reverse primers (*Acaa1b*-AS and *Gapdh*) from selected tissue RNA (Brain, Testis, Kidney, and Liver), then subjected to quantitative RT-PCR. Gene expression level was normalized with *Gapdh*. Primers are listed in Additional file 11.

## Authors' contributions

KN wrote the manuscript, with editing by RS, AK, KA, and HK Microarray design and bioinformatics analyses were performed by KN, YOs, and YOk GRS/A mice were prepared and dissected by NH. HN and NY developed the original viewer for the expression data. KW performed the microarray experiments, and KO performed the *in-situ *hybridization experiments. CK performed quantitative RT-PCR analysis. HK organized and directed the project.

## Supplementary Material

Additional file 1 **Signal intensities from AFAS probes for *Tsix***. AFAS probes designed for *Tsix*, which reflects the abundance of *Xist *RNA, detected expression in the 11 adult mouse tissues (mixed males and females), but not in the testis. Click here for file

Additional file 2 **Antisense expression of mouse imprinted genes**. AFAS probes for several imprinted genes (*Igf2r*, *Kcnq1*, *Gnas*, *Dio3*, and *Ube3a*), which are known to give rise to antisense transcripts, gave prominent signals. Click here for file

Additional file 3 **Numbers of valid probes in adult mouse tissue profiling**. A significantly higher number of AFAS probes than sense probes detected transcripts only within random-primed samples, but not among the oligo-dT primed targets. Click here for file

Additional file 4 **Highest signal intensities from expression profiling of the 12 normal adult tissues**. Probes of 120 genes gave signals with a higher than average intensity according to inter-array normalization. Click here for file

Additional file 5 **Expression analyses of sense and antisense transcripts of *Acaa1b***. Quantitative RT-PCR, ISH and microarray analyses were able to detect this transcript within the testis and kidney. Click here for file

Additional file 6 **List of genes for which expression of the antisense transcript and sense transcript markedly changed in tumors**. Forty-eight were up-regulated and nine were down-regulated within tumor regions, compared with in normal regions. Click here for file

Additional file 7 **Changes in expression of *Pdcd6 *and *Drd4***. Click here for file

Additional file 8 **Selection of target regions for microarray probe design**. Target region selection for the probe design is summarized. Click here for file

Additional file 9 **List of primers for PCR amplification of cDNA fragments to generate probes for Northern blot analysis and *in situ *hybridization**. Primers to amplify probes specific for sense and antisense of *Acaa1b*, *Aard*, and *Thbd *are listed. Click here for file

Additional file 10 **Negative control experiment of *Aard in situ *hybridization**. Click here for file

Additional file 11 **Primers for real-time quantitative RT-PCR**. Primers for real-time quantitative RT-PCR (*Gapdh *and *Acaa1b*-AS) are listed. Click here for file
