# Supplementary material for: Identification of novel endogenous antisense transcripts by DNA microarray analysis targeting complementary strand of annotated genes
Source: BMC Genomics. 2009 Aug 22;10:392. doi: 10.1186/1471-2164-10-392 (PMC2741491; doi:10.1186/1471-2164-10-392)
Supplement: Additional file 1 — Signal intensities from AFAS probes for Tsix. AFAS probes designed for Tsix, which reflects the abundance of Xist RNA, detected expression in the 11 adult mouse tissues (mixed males and females), but not in the testis. [file 1471-2164-10-392-S1.pdf]

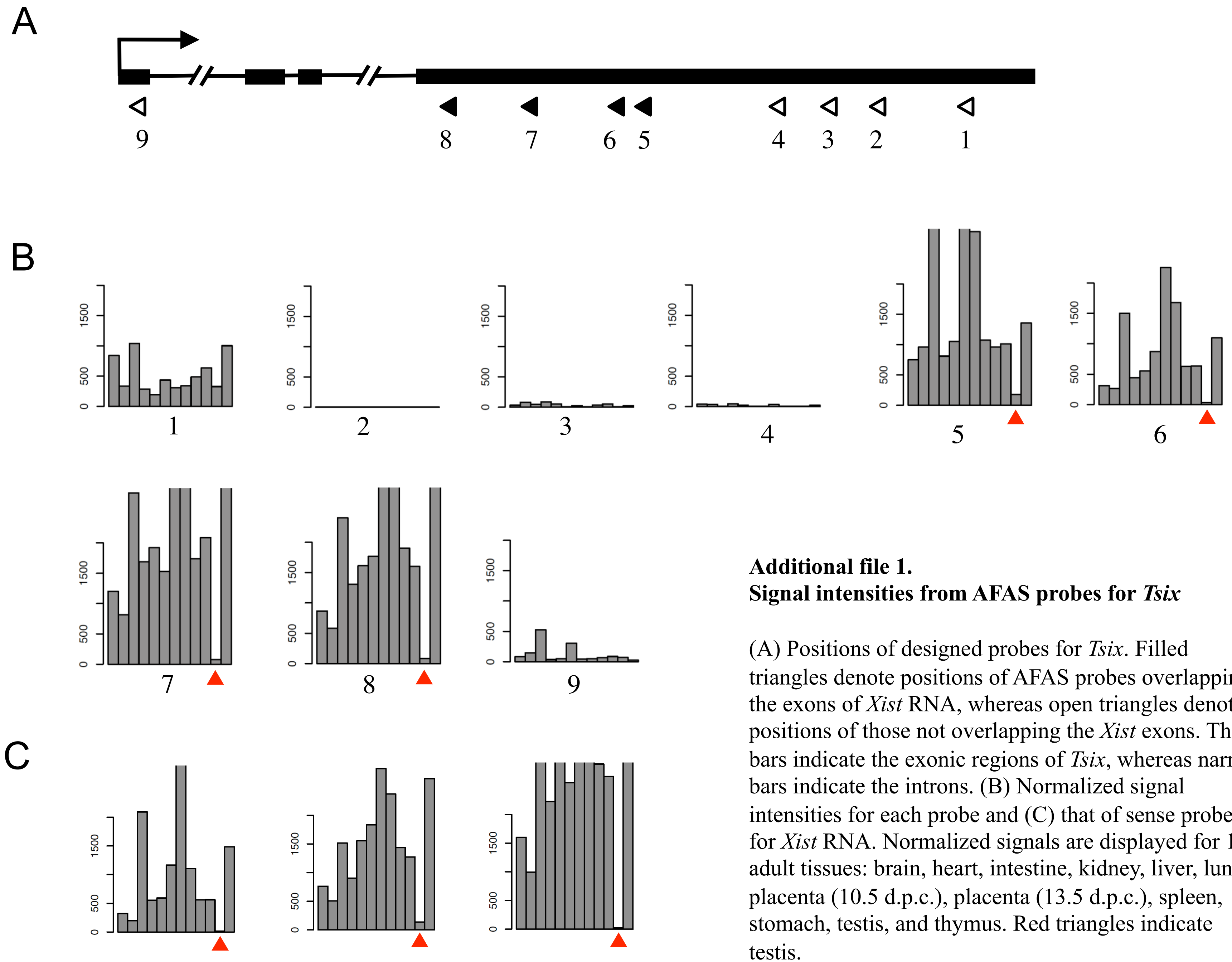

Additional file 1.
Signal intensities from AFAS probes for *Tsix*

(A) Positions of designed probes for *Tsix*. Filled triangles denote positions of AFAS probes overlapping the exons of *Xist* RNA, whereas open triangles denote positions of those not overlapping the *Xist* exons. Thick bars indicate the exonic regions of *Tsix*, whereas narrow bars indicate the introns. (B) Normalized signal intensities for each probe and (C) that of sense probes for *Xist* RNA. Normalized signals are displayed for 12 adult tissues: brain, heart, intestine, kidney, liver, lung, placenta (10.5 d.p.c.), placenta (13.5 d.p.c.), spleen, stomach, testis, and thymus. Red triangles indicate testis.
