# Supplementary material for: Identification of novel endogenous antisense transcripts by DNA microarray analysis targeting complementary strand of annotated genes
Source: BMC Genomics. 2009 Aug 22;10:392. doi: 10.1186/1471-2164-10-392 (PMC2741491; doi:10.1186/1471-2164-10-392)
Supplement: Additional file 2 — Antisense expression of mouse imprinted genes. AFAS probes for several imprinted genes (Igf2r, Kcnq1, Gnas, Dio3, and Ube3a), which are known to give rise to antisense transcripts, gave prominent signals. [file 1471-2164-10-392-S2.pdf]

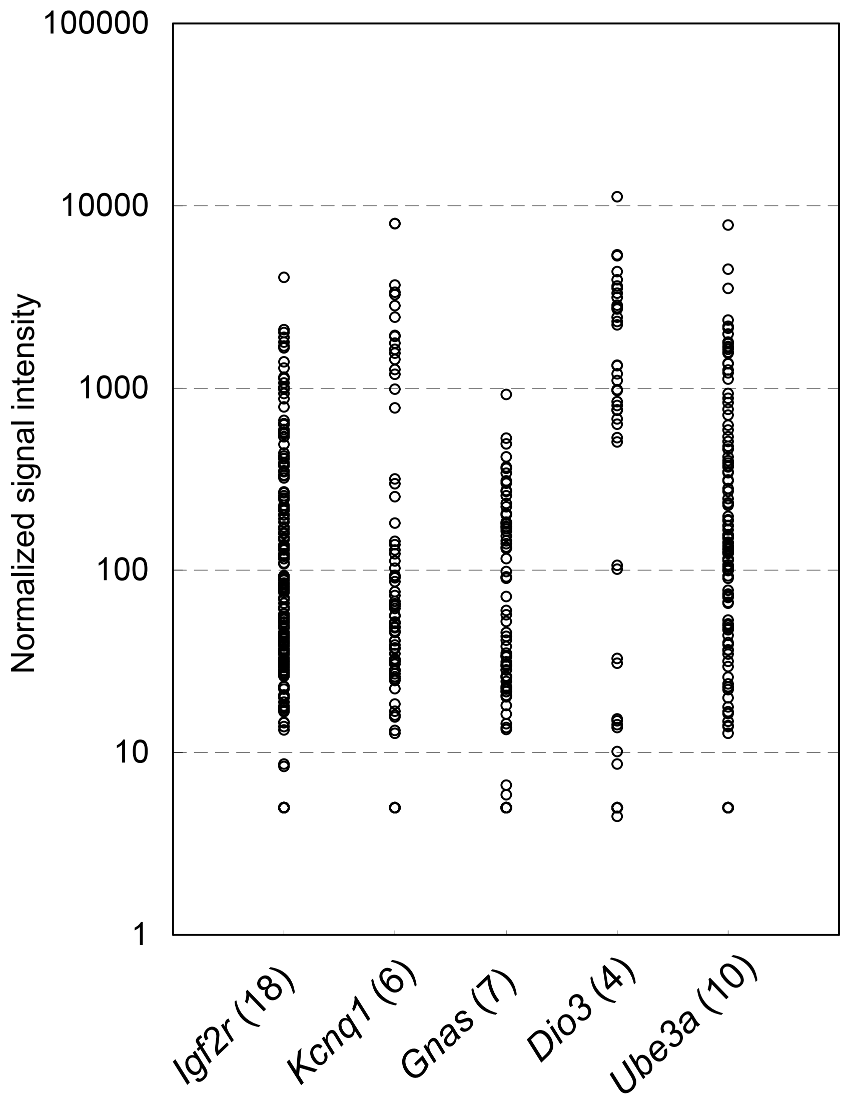

Additional file 2.
Antisense expression of mouse imprinted genes

Normalized signal intensities (normal adult mouse tissues) from AFAS probes corresponding to *Igf2r*, *Kcnq1*, *Gnas*, *Dio3*, and *Ube3a* (antisense transcripts are known for these genes). Numbers of the corresponding AFAS probes are shown in parentheses.
