# Supplementary material for: Identification of novel endogenous antisense transcripts by DNA microarray analysis targeting complementary strand of annotated genes
Source: BMC Genomics. 2009 Aug 22;10:392. doi: 10.1186/1471-2164-10-392 (PMC2741491; doi:10.1186/1471-2164-10-392)
Supplement: Additional file 3 — Numbers of valid probes in adult mouse tissue profiling. A significantly higher number of AFAS probes than sense probes detected transcripts only within random-primed samples, but not among the oligo-dT primed targets. [file 1471-2164-10-392-S3.pdf]

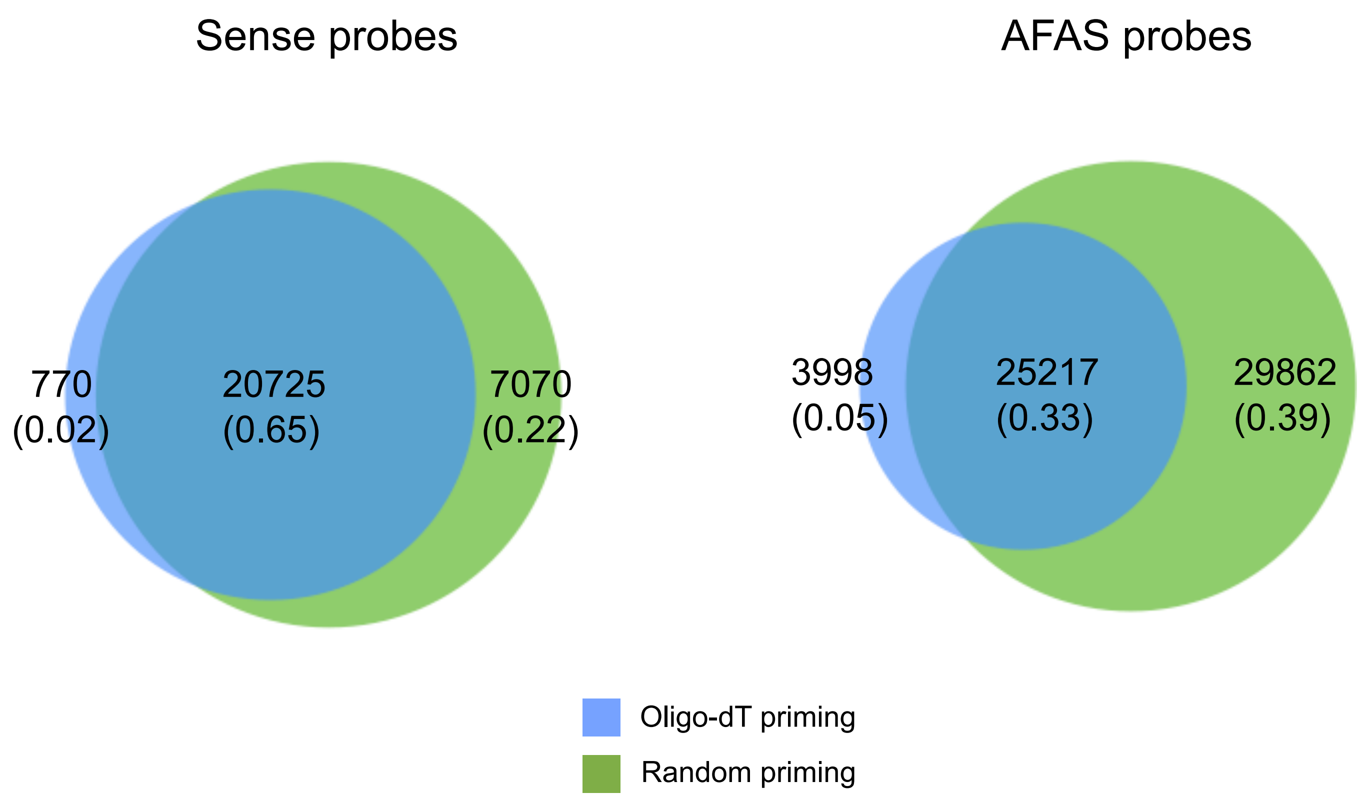

Additional file 3. Numbers of valid probes in adult mouse tissue profiling

Sums of probes (detailed definition is described in Methods section) detected by the random-priming method and by both priming methods in 12 adult mouse tissues are denoted. Detection of the expression for every probe was judged according to the "gIsPosAndSignif" flag in Agilent's FeatureExtraction file. Whereas the ratio (number in the brackets) of antisense probes detected only by random-priming was higher than that of sense probes (P<2.2e-16 by Fisher's exact test), the ratio of antisense probes detected by both priming methods was less than that of the sense probes (P<2.2e-16 by Fisher's exact test).
