# Supplementary material for: Identification of novel endogenous antisense transcripts by DNA microarray analysis targeting complementary strand of annotated genes
Source: BMC Genomics. 2009 Aug 22;10:392. doi: 10.1186/1471-2164-10-392 (PMC2741491; doi:10.1186/1471-2164-10-392)
Supplement: Additional file 4 — Highest signal intensities from expression profiling of the 12 normal adult tissues. Probes of 120 genes gave signals with a higher than average intensity according to inter-array normalization. [file 1471-2164-10-392-S4.pdf]

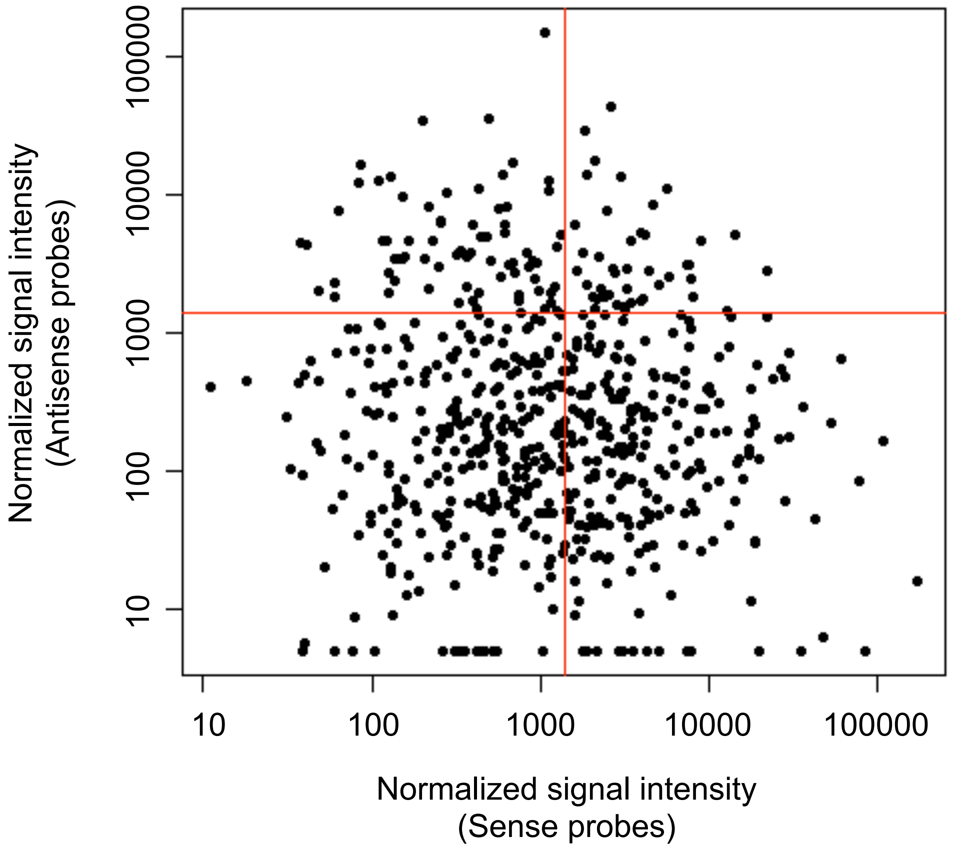

Additional file 4.
Highest signal intensities from expression profiling of the 12 normal adult tissues

Highest signal intensities for both sense and antisense probes for 12 normal adult tissues are plotted. Red line indicates the average signal obtained for the interarray normalization. All signals are derived from hybridizations of random-primed samples.
