# Supplementary material for: Identification of novel endogenous antisense transcripts by DNA microarray analysis targeting complementary strand of annotated genes
Source: BMC Genomics. 2009 Aug 22;10:392. doi: 10.1186/1471-2164-10-392 (PMC2741491; doi:10.1186/1471-2164-10-392)
Supplement: Additional file 5 — Expression analyses of sense and antisense transcripts of Acaa1b. Quantitative RT-PCR, ISH and microarray analyses were able to detect this transcript within the testis and kidney. [file 1471-2164-10-392-S5.pdf]

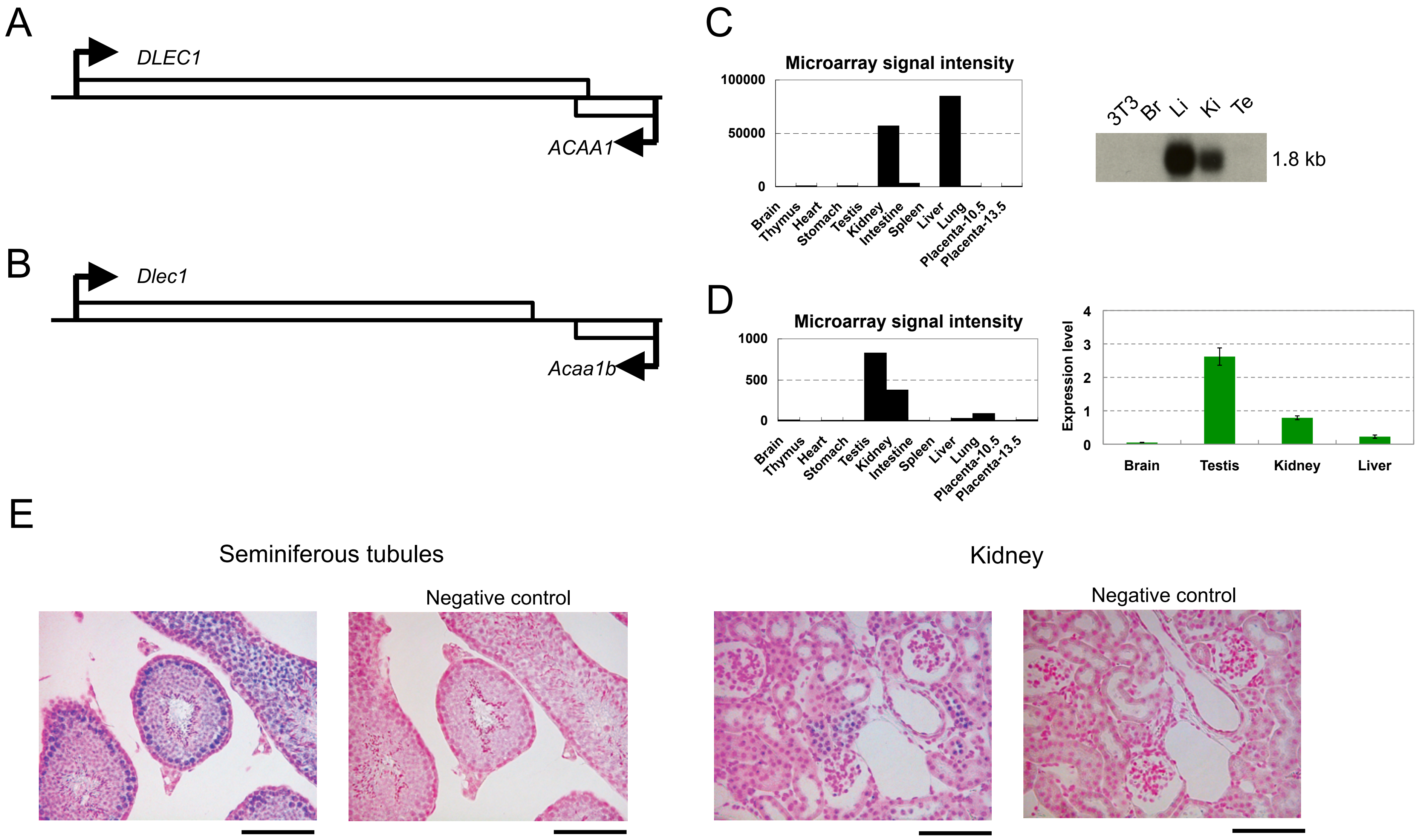


**Additional file 5. Expression analyses of sense and antisense transcripts of *Acaa1b***

(**A**) Schematic illustration of *ACAA1* and *DLEC1* in humans. (**B**) Mouse orthologous partners, *Acaa1b* and *Dlec1*. (**C**) Microarray signals and Northern analysis for sense expression of *Acaa1b* in NIH3T3 cells, brain (Br), liver (Li), kidney (Ki), and testis (Te). (**D**) Microarray signals and RT-PCR analysis for antisense transcript expression. Expression level denotes normalized gene expression level with *Gapdh*. (**E**) *in-situ* hybridization experiment results for antisense expression in seminiferous tubules and kidney. Negative control experiments used probe sequence selected from *Oryza sativa* putative leaf protein (NM_197207). Scale bars: 100 µm.
