# Supplementary material for: Identification of novel endogenous antisense transcripts by DNA microarray analysis targeting complementary strand of annotated genes
Source: BMC Genomics. 2009 Aug 22;10:392. doi: 10.1186/1471-2164-10-392 (PMC2741491; doi:10.1186/1471-2164-10-392)
Supplement: Additional file 6 — List of genes for which expression of the antisense transcript and sense transcript markedly changed in tumors. Forty-eight were up-regulated and nine were down-regulated within tumor regions, compared with in normal regions. [file 1471-2164-10-392-S6.pdf]

| Quadrant | RefSeq ID | Gene annotation |
|---|---|---|
| Right upper | NM_013649 | receptor-like tyrosine kinase (Ryk), transcript variant 1 |
| Right upper | NM_010444 | nuclear receptor subfamily 4, group A, member 1 (Nr4a1) |
| Right upper | NM_007395 | activin A receptor, type 1B (Acvr1b) |
| Right upper | NM_009874 | cyclin-dependent kinase 7 (homolog of Xenopus MO15 cdk-activating kinase) (Cdk7) |
| Right lower | NM_009764 | breast cancer 1 (Brca1) |
| Right lower | NM_022310 | heat shock protein 5 (Hspa5) |
| Right lower | NM_008506 | v-myc myelocytomatosis viral oncogene homolog 1, lung carcinoma derived (avian) (Mycl1) |
| Right lower | NM_007783 | c-src tyrosine kinase (Csk) |
| Right lower | NM_008871 | serine (or cysteine) peptidase inhibitor, clade E, member 1 (Serpine1) |
| Right lower | NM_011729 | excision repair cross-complementing rodent repair deficiency, complementation group 5 (Ercc5) |
| Right lower | NM_011385 | ski sarcoma viral oncogene homolog (avian) (Ski) |
| Right lower | NM_007395 | activin A receptor, type 1B (Acvr1b) |
| Right lower | NM_009397 | tumor necrosis factor, alpha-induced protein 3 (Tnfaip3) |
| Right lower | NM_009983 | cathepsin D (Ctsd) |
| Right lower | NM_013520 | FMS-like tyrosine kinase 3 ligand (Flt3l) |
| Right lower | NM_011051 | programmed cell death 6 (Pdcd6) |
| Left upper | NM_011630 | nuclear receptor subfamily 2, group C, member 2 (Nr2c2) |
| Left lower | NM_021283 | interleukin 4 (Il4) |
| Left lower | NM_009755 | bone morphogenetic protein 1 (Bmp1) |

**Additional file 6. List of genes for which expression of the antisense transcript and sense transcript markedly changed in tumors**

Quadrants correspond to those in **Figure 5A**. Right upper: sense and antisense transcripts were up-regulated in tumors; right lower: antisense transcripts were down-regulated and sense transcripts were up-regulated; left upper: antisense transcripts were up-regulated and sense transcripts were down-regulated; left lower: both sense and antisense transcripts were down-regulated.
