# Supplementary material for: Identification of novel endogenous antisense transcripts by DNA microarray analysis targeting complementary strand of annotated genes
Source: BMC Genomics. 2009 Aug 22;10:392. doi: 10.1186/1471-2164-10-392 (PMC2741491; doi:10.1186/1471-2164-10-392)
Supplement: Additional file 7 — Changes in expression of Pdcd6 and Drd4. [file 1471-2164-10-392-S7.pdf]

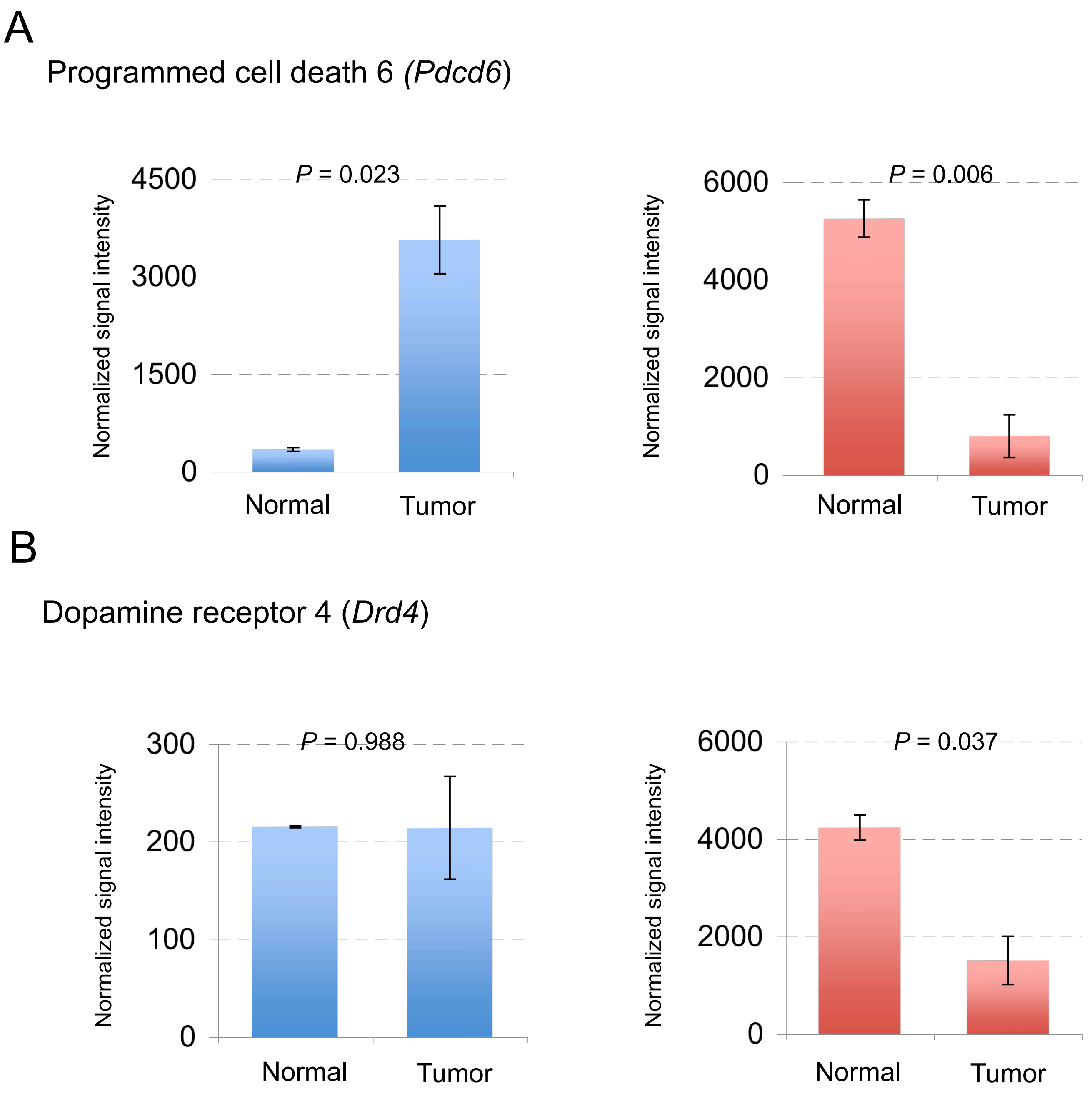

Additional file 7.
Changes in expression of *Pdcd6* and *Drd4*

(**A**) Signal intensities of *Pdcd6* for which sense (blue bars) and antisense (red bars) transcripts were inversely correlated between normal and tumor cells. (**B**) Signal intensities for *Drd4,* for which expression of the antisense transcript (red bars) was markedly changed in tumor cells, whereas that of the sense transcipt (blue bars) was not.
