# Supplementary material for: Identification of novel endogenous antisense transcripts by DNA microarray analysis targeting complementary strand of annotated genes
Source: BMC Genomics. 2009 Aug 22;10:392. doi: 10.1186/1471-2164-10-392 (PMC2741491; doi:10.1186/1471-2164-10-392)
Supplement: Additional file 8 — Selection of target regions for microarray probe design. Target region selection for the probe design is summarized. [file 1471-2164-10-392-S8.pdf]

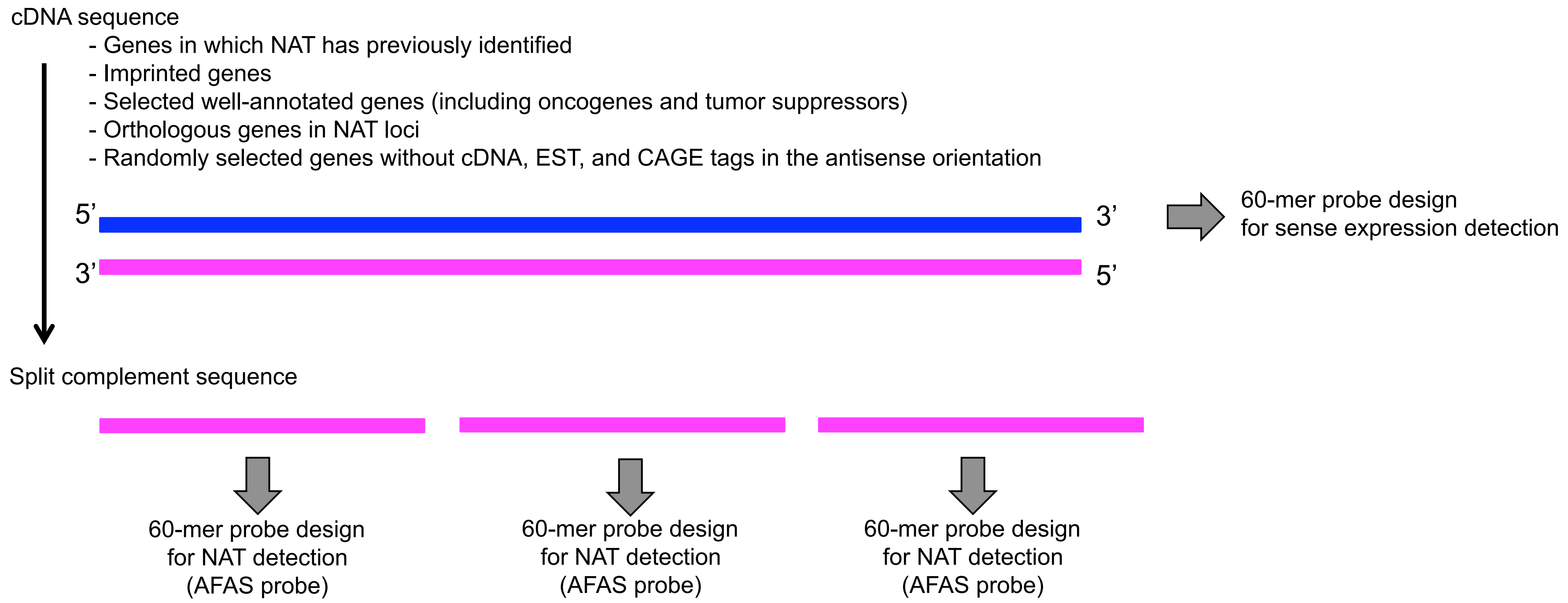

Additional file 8.
Selection of target regions for microarray probe design

Target selection procedure for microarray-probe-design is illustrated. Full-length cDNA sequences were obtained for the design of probes to detect sense gene expression. To make the target sequences to generate the probes for NAT detection (AFAS probes), we initially made complement of the cDNA sequences and then split them into 500 base-pairs fragments. Each fragment was assigned to the probe design program, OligoWiz [1]. For the orthologous genes in NAT loci, only one AFAS probe was designed for each gene (i.e., not split into fragments). Control genes were randomly selected and without cDNA, EST, and CAGE tags in the antisense orientation. Two AFAS probes were designed per control gene. Probes summarized in the table were loaded onto the Agilent 44K custom microarray platform.

[1] Wernersson R, Nielsen HB: OligoWiz 2.0–integrating sequence feature annotation into the design of microarray probes. Nucleic Acids Res 2005, 33(Web Server issue):W611-615.

Category	Number of sense probes	Number of AFAS probes	Total
Known-NAT	48	112	160
Imprinted genes	87	465	552
Selected genes	404	1752	2156
Orthologous genes in NAT loci	648	635	1283
Control genes	1462	2884	4346
Probes unrelated to this work	–	–	33243
