# Supplementary material for: Identification of novel endogenous antisense transcripts by DNA microarray analysis targeting complementary strand of annotated genes
Source: BMC Genomics. 2009 Aug 22;10:392. doi: 10.1186/1471-2164-10-392 (PMC2741491; doi:10.1186/1471-2164-10-392)
Supplement: Additional file 9 — List of primers for PCR amplification of cDNA fragments to generate probes for Northern blot analysis and in situ hybridization. Primers to amplify probes specific for sense and antisense of Acaa1b, Aard, and Thbd are listed. [file 1471-2164-10-392-S9.pdf]

Gene symbol	Accession	Direction	Forward primer	Reverse primer
Acaa1b	NM_146230	Sense	GGCATCCAATCCGGTTCTCTC	TGCCCATCACATCCACCATTC
		Antisense	AGTTTTCAGGGCCACCACCAG	GCATCCAATCCGGTTCTCTCG
Aard	NM_175503	Sense	AAGAAGAGGCAGGACCAGGAG	ATCGAGGGTATGGGTGGAATC
		Antisense	GAGGGTATGGGTGGAATCTAG	TATTAGTCGAAGGGAGGAGGG
Thbd	NM_009378	Sense	AAGAATTGGGACCTCGCTTGG	TGTACATGTGACGTCATAAGC
		Antisense	TTCAAGTCCTCCCTACCCTCG	CAGTGCAGTCAAGGCGAATGC

Additional file 9. List of primers for PCR amplification of cDNA fragments to generate probes for Northern blot analysis and *in situ* hybridization
