# Supplementary material for: Identification of novel endogenous antisense transcripts by DNA microarray analysis targeting complementary strand of annotated genes
Source: BMC Genomics. 2009 Aug 22;10:392. doi: 10.1186/1471-2164-10-392 (PMC2741491; doi:10.1186/1471-2164-10-392)
Supplement: Additional file 10 — Negative control experiment of Aard in situ hybridization. [file 1471-2164-10-392-S10.pdf]

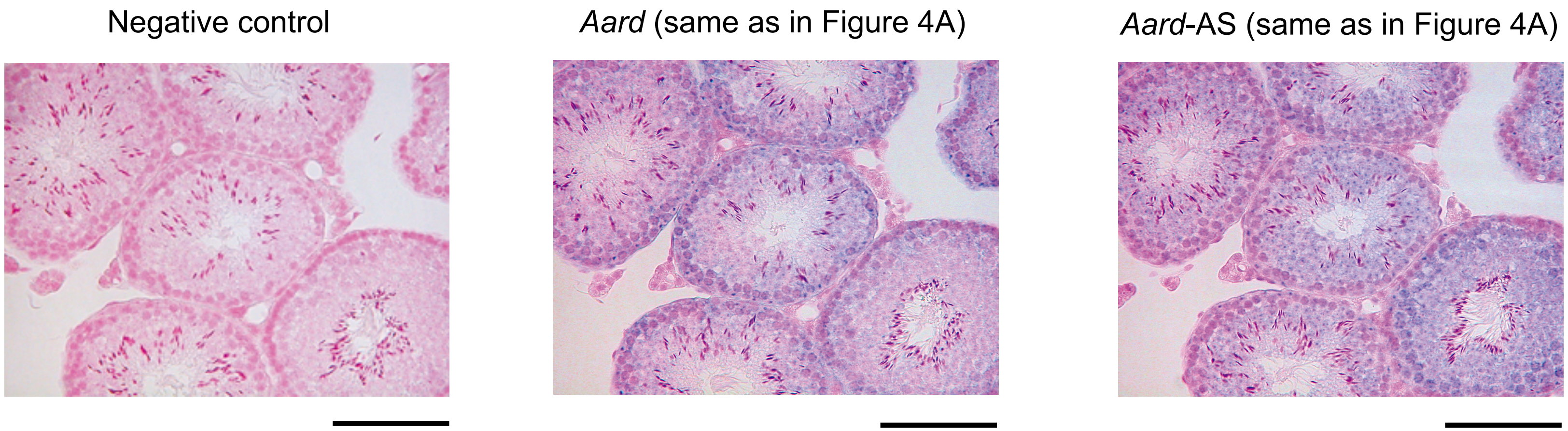


**Additional file 10. Negative control experiment of *Aard in situ* hybridization**
Probe sequence of negative control experiment was selected from *Oryza sativa* putative leaf protein (NM_197207).
Scale bars: 100µm.
