# Supplementary material for: Identification of novel endogenous antisense transcripts by DNA microarray analysis targeting complementary strand of annotated genes
Source: BMC Genomics. 2009 Aug 22;10:392. doi: 10.1186/1471-2164-10-392 (PMC2741491; doi:10.1186/1471-2164-10-392)
Supplement: Additional file 11 — Primers for real-time quantitative RT-PCR. Primers for real-time quantitative RT-PCR (Gapdh and Acaa1b-AS) are listed. [file 1471-2164-10-392-S11.pdf]

Acaa1b-AS	Forward	CCCATCACATCCACCATTCA
	Reverse	GTAATTGCAGCATGGGTACACG
Gapdh	Forward	GTGTTCCTACCCCCAATGTGTC
	Reverse	GGTCCTCAGTGTAGCCCAAGAT

Additional file 11. Primers for real-time quantitative RT-PCR
